# Pretreatment with *Lactobacillus reuteri* F-9-35 attenuates ethanol-induced gastric injury in rats

**DOI:** 10.29219/fnr.v62.1469

**Published:** 2018-10-29

**Authors:** Mao-Cheng Sun, Ping-Ping Hou, Xin-Yu Wang, Chang-Hui Zhao, Bi-Jun Cheng, Yan-Ling Wang, Hong-Wei Hao, Tie-Hua Zhang, Hai-Qing Ye

**Affiliations:** 1 College of Food Science and Engineering, Jilin University, Changchun, China; 2 School of Public Health, Jilin Medical University, Jilin City, China; 3 School of Pharmaceutical Sciences, Jilin University, Changchun, China; 4 Fullarton Bioengineering Technology Co., Ltd., Beijing, China

**Keywords:** Lactobacillus reuteri, ethanol, gastric injury, inflammatory, antioxidant, NF-κB pathway

## Abstract

**Background:**

Previous studies suggested that probiotics intervention may be one of the methods for preventing and/or treating gastric ulcer.

**Objective:**

The aim of the study was to compare the preventive effects of a spaceflight mutant *Lactobacillus reuteri* F-9-35 and its wild type on ethanol-induced gastric injury in rats.

**Design:**

Forty rats were randomly allocated into five groups: a normal group (NOR), ethanol group (EtOH), skim milk group (MILK), *L. reuteri* F-9-35 group (F935) and wild-type group (WT). The NOR and EtOH groups received 1 ml of distilled water by daily gavage for 14 days. The MILK group received 1 ml of skim milk alone, while the F935 and WT groups were administered 1 ml of skim milk containing the mutant and wild type (1 × 10^10^ colony-forming unit/ml) by daily gavage for 14 days, respectively. Acute gastric injury was induced by absolute alcohol 1 h after the final administration of different treatments, except for the NOR group.

**Results:**

Pretreatment with *L. reuteri* F-9-35, but not milk alone or milk with the *L. reuteri* wild type, showed significant reduction of ethanol-induced gastric injury, as evidenced by lowering of ulcer index, ulcer area (%), and histological lesion. F-9-35 decreased the levels of lipid peroxidation and myeloperoxidase and increased mucus, glutathione, and nitric oxide levels in gastric tissue. Moreover, F-9-35 inhibited the expression of proinflammatory genes including gastric tumor necrosis factor-α, interleukin-1β, and cyclooxygenase-2 and decreased the activity of nuclear factor kappa B (NF-κB).

**Conclusion:**

These findings indicated that *L. reuteri* F-9-35 pretreatment can attenuate ethanol-induced gastric injury in rats by inhibiting oxidative stress and inflammatory response. Together, *L. reuteri* F-9-35 has potential preventive efficacy on gastric ulcer.

Alcohol is considered a part of social culture in many countries and is associated with numerous social behaviors, such as party time and celebrations (1). However, the consumption of large amounts of ethanol can directly lead to acute gastric mucosal bleeding, edema, and erosion (2). Long-term drinking has been reported to be associated with gastric mucosal lesions including gastric ulcer and gastritis (3). Although the mechanism of alcoholic gastric damage has not been fully elucidated, accumulating evidence has proved that oxidative stress and inflammatory response are involved in the occurrence and development of gastric mucosal lesions (4, 5).

It is well known that probiotics have many health effects on the host when consumed, especially on the gastrointestinal tract (6). Increasing evidence in animal models also suggests that probiotics are promising for preventing and/or treating gastric ulcers. *Lactobacillus rhamnosus* GG pretreatment attenuates acetic acid-induced gastric ulcer in rats by the regulation of expression of various growth factors (7), as well as ethanol-induced acute gastric mucosa injury by increasing mucosal prostaglandin (PG) E2 level and mucin mRNA expression (8). Treatment of rats with the probiotic mixture VSL #3^®^ (eight probiotic bacteria) heals gastric ulcer induced by acetic acid through the inhibition of pro-inflammatory response and the improvement of vascular regenerative protein expression (9). *Lactobacillus plantarum* LC27 and *Bifidobacterium longum* LC67 not only alleviate ethanol-induced gastric injury but also ameliorate ethanol-induced hepatic injury in mice (10).

*Lactobacillus reuteri*, one species of *Lactobacillus*, is a candidate for probiotics because it may be able to prevent or alleviate a variety of human diseases, such as early-life disorders, obesity, and enteric infection (11). However, there is no data reported on gastroprotective activity of *L. reuteri*. Therefore, we examined the effect of a spaceflight mutant *L. reuteri* F-9-35 and its wild type on gastric injury induced by ethanol. The effects of two strains on ulcer index (UI), the percentage of ulcer area (UA), histological lesion, and the levels of malondialdehyde (MDA), myeloperoxidase (MPO), glutathione (GSH), nitric oxide (NO), mucus, and the expression of tumor necrosis factor-α (TNF-α), interleukin-1β (IL-1β), cyclooxygenase-2 (COX-2) mRNA, and nuclear factor kappa B (NF-κB) activity in gastric tissue was studied. In addition, hepatic MDA, triglycerides (TG) and GSH levels were also determined.

## Materials and methods

### Bacterial strains

The mutant *L. reuteri* F-9-35 and its wild type *L. reuteri* GS-23 were provided by Fullarton Bioengineering Technology Co., Ltd. (Beijing, China). The wild type was sent into outer space by China’s Shenzhou-11 spacecraft in 2016 for 31 days and 18.5 h of space flight. After the spacecraft landed, one mutant *L. reuteri* F-9-35 was chosen for this study because it had better gastrointestinal resistance and hydrophobicity than its wild type *in vitro*, and the safety evaluation indicated that the mutant was safe (12).

### Preparation of L. reuteri for rat feeding

Two strains were each inoculated in deMan Rogosa and Sharpe (MRS) broth and incubated for 18 h at 37°C. The cells were adjusted to a concentration of 1 × 10^10^ colony-forming unit (CFU)/mL. The pellets were obtained by centrifugation (6,000 g, 10 min, 4°C), washed in 0.85% NaCl solution twice, and then resuspended in 12% (w/v) skim milk sterilized and stored at –80°C until further use.

### Animal study design

Forty female Wistar rats (180–220 g) were obtained from Yisi Experimental Animal Technology Co., Ltd. (Changchun, China). Rats were housed in an air-conditioned room at 22 ± 2°C and had free access to tap water and a standard diet. After acclimation for 7 days, rats were randomly divided into five groups (*n* = 8): a normal group (NOR), ethanol group (EtOH), skim milk group (MILK), wild type group (WT), and F-9-35 group (F935). The NOR and EtOH groups were administered 1 mL/day distilled water by daily gavage, while the MILK, WT, and F935 groups received 12% sterilized skim milk, with or without the wild type or the mutant (1 × 10^10^ CFU/mL) for 14 days. At Day 13, all rats were fasted but not of water for 24 h after being gavaged. Then gastric ulcer was induced by intragastric absolute ethanol (5 ml/kg BW) 1 h after the final administration of probiotics (13, 14). An hour after ethanol treatment, rats were sacrificed under anesthesia after injection of sodium pentobarbital (60 mg/kg BW). The stomach was excised and cut open along the greater curvature, cleaned with cold normal saline and macroscopically evaluated for UI scoring as described previously (15). Each stomach was photographed for determination of UA percentage by ImageJ software (1.51j, National Institutes of Health, Bethesda, USA). Then each stomach was dichotomized, and 1 × 1 cm glandular segments from one moiety was fixed in 10% neutral formalin buffer for histopathologic analysis; the remaining glandular part was weighed and immersed in 0.1% Alcian blue 8GX (Yuanye, Shanghai, China) solution for the determination of gastric wall mucus. The other moiety and liver sample were immediately frozen in liquid nitrogen for 3 h and transferred to −80°C for further determination. The protocol was approved by the Institutional Animal Care and Use Committee of Jilin University.

### Histological examination

Gastric sections fixed by formalin were embedded in paraffin. Full-thickness sections (5 μm) were stained with hematoxylin and eosin. The histological damage was evaluated and scored by a pathologist observer in a blinded manner as described previously (16).

### Gastric wall mucus determination

Mucus content was measured by the spectrophotometric method as previously described (17).

### Biochemical analysis

The levels of MPO, MDA, GSH, and NO in gastric tissue and MDA, GSH, and TG in hepatic tissue were determined using commercial kits (Jiancheng Bioengineering Institute, Nanjing, China) according to the manufacturer’s instructions. Total protein content was measured with a Bradford Protein Assay Kit (Beyotime, Shanghai, China).

### Real-time quantitative PCR

Real-time quantitative PCR (qPCR) was carried out to assess changes in the expression of mRNA for TNF-α, IL-1β, and COX-2. Total RNA was extracted from gastric tissue using TRIzol Reagent (Thermo Fisher Scientific, Waltham, USA) according to the manufacturer’s protocol. RNA was reverse-transcribed to cDNA with an All-In-One RT 5X MasterMix kit containing a genomic DNA removal procedure (Applied Biological Materials, Richmond, Canada). The qPCR was performed using the EvaGreen 2X qPCR MasterMix (Applied Biological Materials) on a Bio-Rad CFX96 Real-Time instrument. The qPCR primers used were as follows: COX-2 forward, GGTTCACCCGAGGACTGGGC, and reverse, CGCAGGTGCTCAGGGACGTG (9); TNF-α forward, TGTGCCTCAGCCTCTTCTCATTCA, and reverse, CATTTGGGAACTTCTCCTCCTTG; IL-1β forward, AATGACCTGTTCTTTGAGGCTGAC and reverse, CGAGATGCTGCTGTGAGATTTGAAG; GAPDH forward, TGCTGGTGCTGAGTATGTCGTG, and reverse, CGGAGATGATGACCCTTTTGG (18). The gene expression level was calculated using the ΔΔCt method and normalized to GAPDH as an internal control.

### Western blot analysis

The gastric tissues were hand-homogenized in liquid N_2_ and the total proteins were extracted by a cold lysis buffer with protease and phosphatase inhibitors (Beyotime, Shanghai, China). The protein content was measured using a BCA Protein Assay Kit (Beyotime). Proteins were separated by 12% sodium dodecyl sulfate–polyacrylamide gels and then transferred onto a PVDF membrane. The membrane was blocked for 2 h with 3% bovine serum albumin in TBST buffer (Tris-buffered saline and 0.1% Tween 20) at room temperature and then incubated overnight with primary antibodies against either β-actin, NF-κB p-65 (Proteintech, Chicago, USA), or NF-κB p-p65 (Arigo, Hsinchu City, China) at 4°C and subsequently with secondary antibody HRP-conjugated goat anti-rat IgG H&L (Proteintech) for 2 h. Specific bands were detected using a chemiluminescent Western blot imaging system (Azure, Dublin, USA).

### Statistical analysis

Data were presented as mean ± SD. A Kruskal–Wallis test with Dunn’s correct was performed using GraphPad Prism 6.0 (GraphPad Software Inc., San Diego, USA). Statistical significance was set at *p* < 0.05.

## Results

### Stomach appearances of rats

**Fig. 1:**
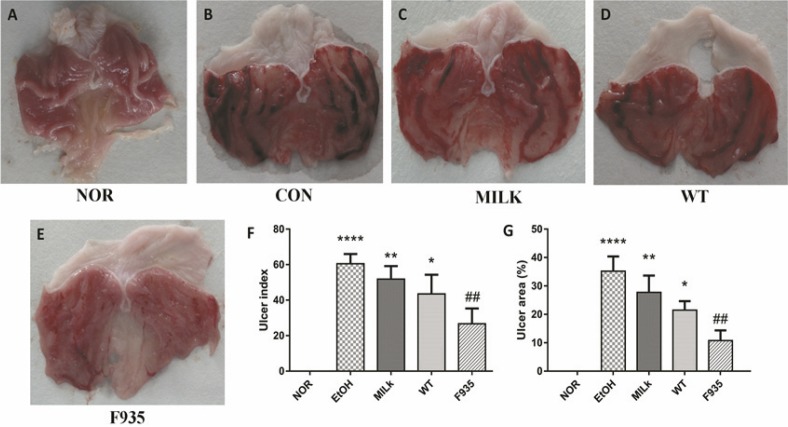
Macroscopic evaluation of gastric injury. (A) Normal (NOR) group; (B) ethanol (EtOH) group; (C) skim milk (MILK) group; (D) wild-type (WT) group; (E) *L. reuteri* F-9-35 (F935) group; (F) ulcer index; (G) ulcer area (%). ****p < 0.0001, **p < 0.01, and *p < 0.05 vs. the NOR group; ##*p* < 0.01 vs. the EtOH group.

The stomach photos after ethanol induction were shown in Fig. 1A through E. The results showed the stomachs from the EtOH group had severe bleeding and ulcer. Although pretreatment with milk alone or two stains relieved gastric damage compared to the EtOH group, the F935 group showed the smallest injury. As shown in Fig. 1F and G, compared with the EtOH group, the UI and UA (%) in the F935 group were significantly decreased (UI, 26.67 ± 8.62 vs. 60.50 ± 5.47, *p* < 0.01; UA, 10.75 ± 3.60 vs. 35.19 ± 5.21%, *p* < 0.01), while there were no significant differences in either the MILK or WT group (*p* > 0.05).

### Histological evaluation of gastric injury

**Fig. 2:**
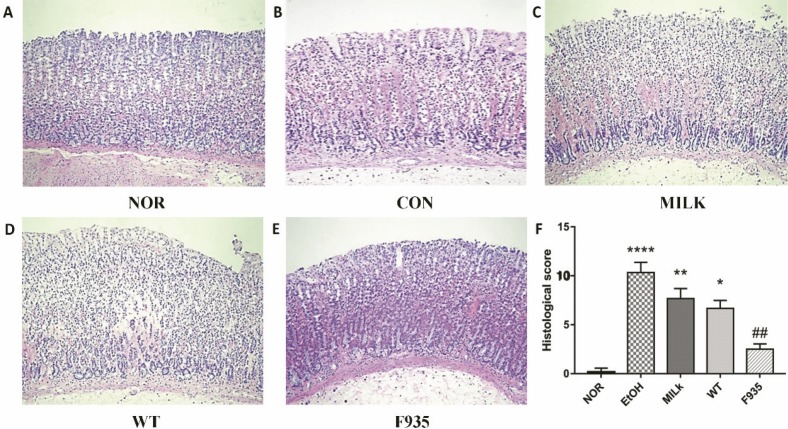
Histopathological analysis of gastric mucosa (hematoxylin and eosin staining, ×100). (A) NOR group; (B) EtOH group; (C) MILK group; (D) WT group; (E) F935 group; (F) histological score. ****p < 0.0001, **p < 0.01, and *p < 0.05 vs. the NOR group, ##*p* < 0.05 vs. the EtOH group.

Histological evaluation (Fig. 2) showed the EtOH group (score: 10.33 ± 1.03) exhibited severe pathological changes including epithelial damage, glandular damage, massive bleeding, and inflammatory cell infiltration. The MILK group (score: 7.67 ± 1.00) and WT group (score: 6.67 ± 0.82) showed mild gastric mucosa injury compared to the EtOH group. Notably, minimal damage was observed in the F935 group (score: 2.50 ± 0.55).

### Gastric wall mucus

**Fig. 3:**
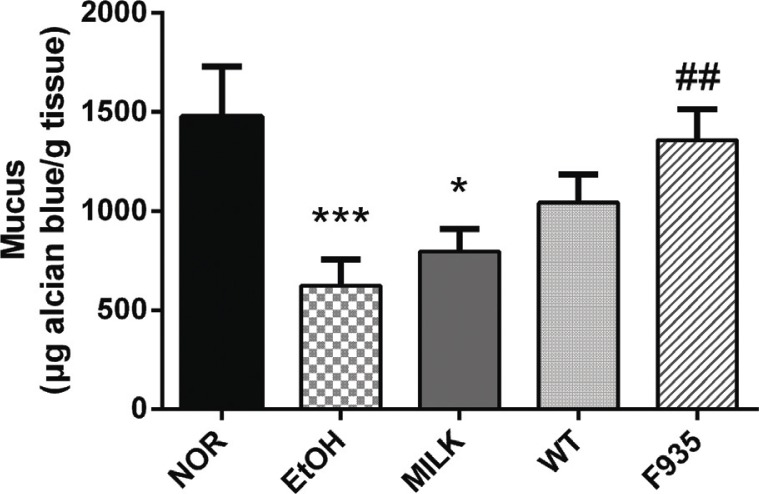
Effects of *L. reuteri* on gastric wall mucus. ***p < 0.001 and *p < 0.01 vs. the NOR group; ##*p* < 0.01 vs. the EtOH group.

The mucus content in different treatment groups is shown in Fig. 3. The mucus content was significantly decreased in the EtOH group compared to the NOR group (*p* < 0.001). Compared with the EtOH group, *L. reuteri* F-9-35 significantly recovered the mucus content (*p* < 0.01), while animals treated with milk alone or wild type showed no significant difference (*p* > 0.05).

### The levels of MPO, MDA, GSH, and NO in gastric tissue

**Fig. 4:**
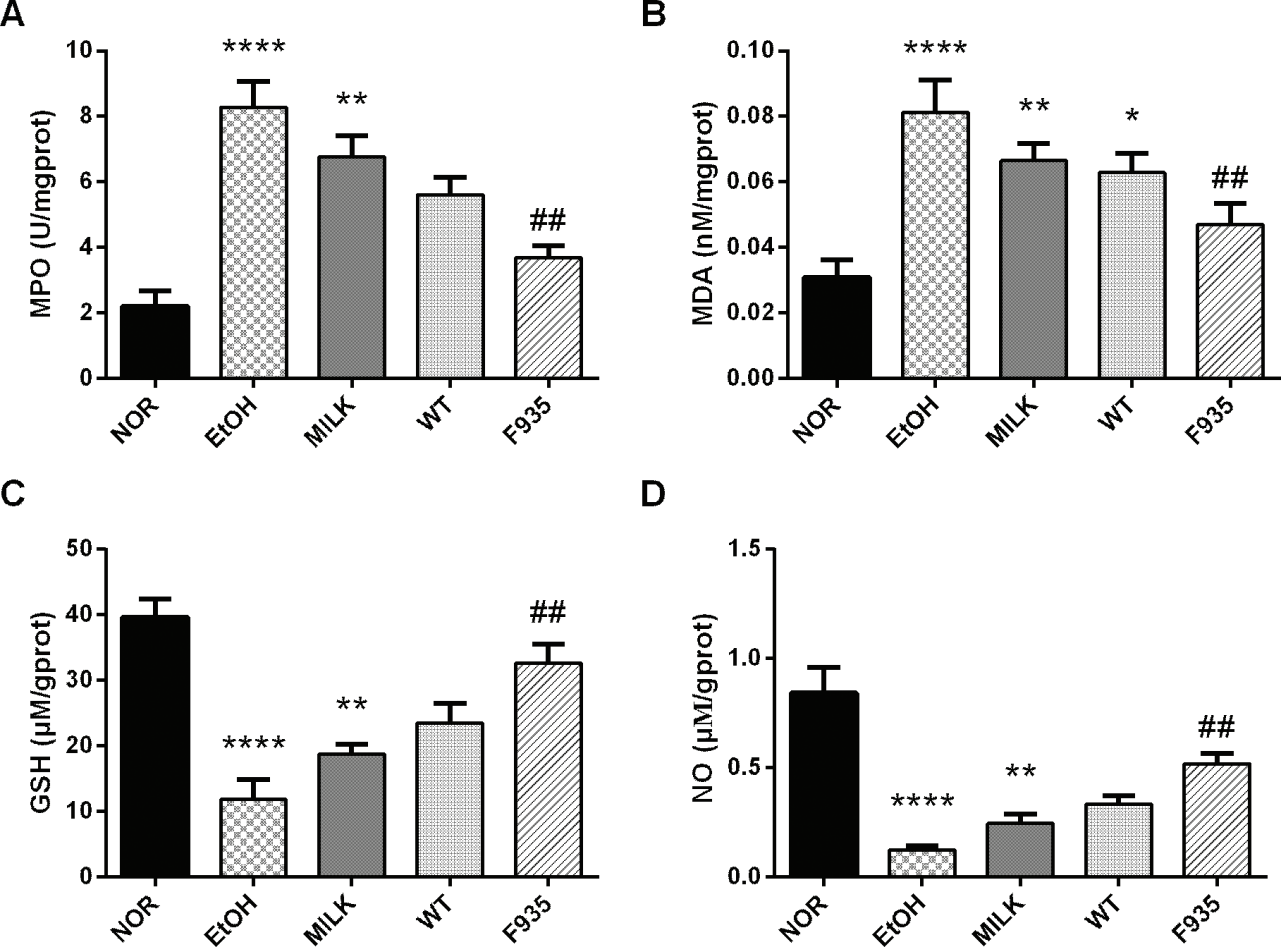
Effects of *L. reuteri* on the levels of MPO, MDA, GSH, and NO in gastric tissue. (A) MPO; (B) MDA; (C) GSH; (D) NO. ****p < 0.0001, **p < 0.01, and *p < 0.05 vs. the NOR group; ##*p* < 0.01 vs. the EtOH group. MPO, myeloperoxidase; MDA, malondialdehyde; GHS, glutathione; NO, nitric oxide.

As shown in Fig. 4, ethanol treatment significantly increased MPO and MDA levels (*p* < 0.0001) and reduced GSH and NO levels (*p* < 0.0001) compared to the NOR group. The GSH, NO, MPO, and MDA levels in the MILK and WT groups had no significant difference compared to the EtOH group (*p* > 0.05). After pretreatment with *L. reuteri* F-9-35, the GSH and NO levels were markedly improved compared to the EtOH group (*p* < 0.01), while the MPO and MDA levels were clearly reduced (*p* < 0.01).

### The relative expression of TNF-α, IL-1β, and COX-2 mRNA in gastric tissue

**Fig. 5:**
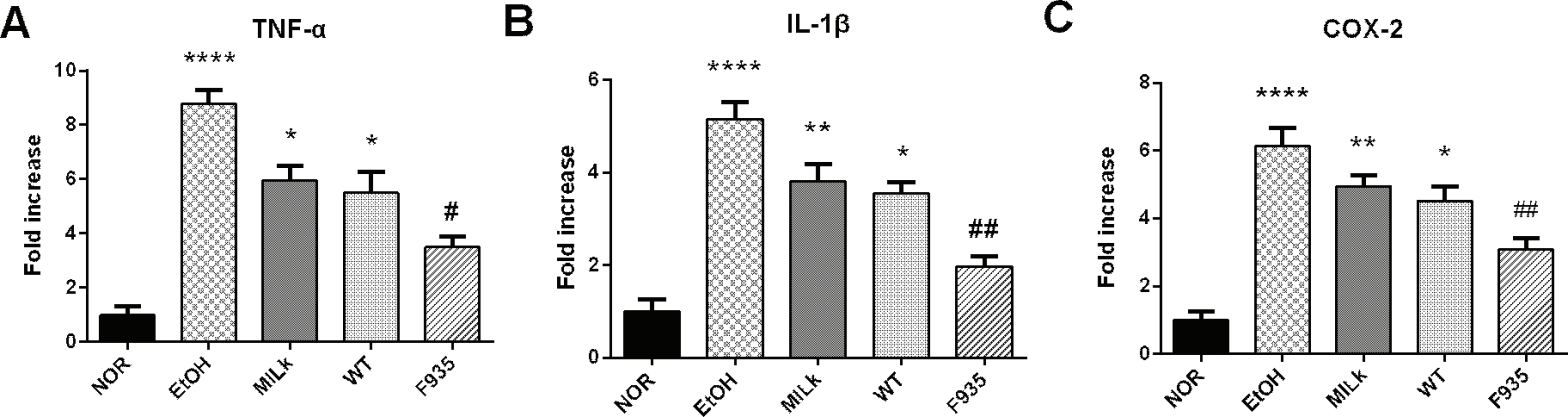
Effects of *L. reuteri* on the expressions of TNF-α, IL-1β, and COX-2 mRNA in gastric tissue. (A) TNF-α; (B) IL-1β; (C) COX-2. ****p < 0.0001, **p < 0.01, and *p < 0.05 vs. the NOR group; ##*p* < 0.01 and #*p* < 0.05 vs. the EtOH group. TNF-α, tumor necrosis factor-α; IL-1β, interleukin-1β; COX-2, cyclooxygenase-2.

As shown in Fig. 5, the EtOH group showed higher expression of TNF-α, IL-1β, and COX-2 mRNA (8.79, 5.15, and 6.13 times the NOR group values, respectively). Expression of these genes in the MILK and WT groups decreased, but there were no significant differences compared to the EtOH group (*p* > 0.05). However, *L. reuteri* F-9-35 pretreatment significantly suppressed the overexpression of these genes induced by ethanol.

### Activation of the NF-κB pathway in gastric tissue

**Fig. 6:**
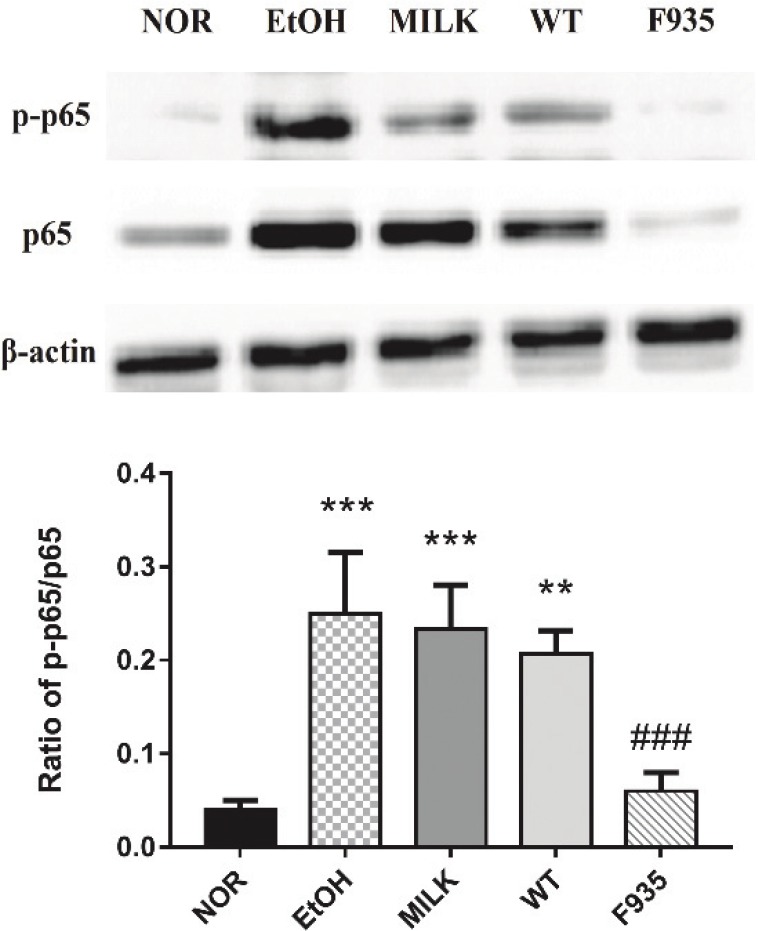
Effects of *L. reuteri* on NF-κB activity in gastric tissue. ***p < 0.001 and **p < 0.01 vs. the NOR group; ###*p* < 0.001 vs. the EtOH group.

The ratio of p-p65 to p65 can be used to reflect the activity of the NF-κB pathway (10). As shown in Fig. 6, ethanol treatment significantly increased NF-κB activity in the EtOH, MILK, and WT groups compared to the NOR group (*p* < 0.001, *p* < 0.001, and *p* < 0.01, respectively). However, the NF-κB activity of gastric tissues from F935 rats was significantly downregulated compared to the EtOH group (*p* < 0.001).

**Fig. 7:**
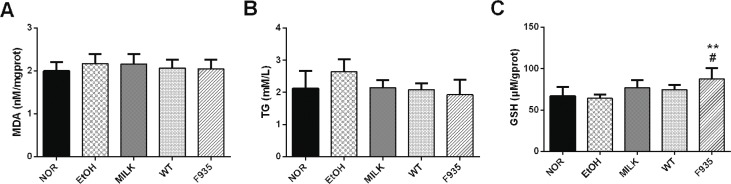
Effects of *L. reuteri* on the levels of MDA, TG, and GSH in liver sample. (A) MDA; (B) TG; (C) GSH. **p < 0.01 vs. the NOR group; #*p* < 0.05 vs. the EtOH group. MDA, malondialdehyde; TG, triglycerides; GHS, glutathione.

### Hepatic MDA, TG, and GSH levels

The MDA and TG levels in the liver had no significant difference among all groups (Fig. 6A and B, *p* > 0.05). Of note, the GSH level in the F935 group was significantly higher than that of the NOR or EtOH groups (Fig. 6C; *p* < 0.01 and *p* < 0.05, respectively).

## Discussion

Exposure of high concentration ethanol in the stomach can result in severe gastric ulcer with bleeding by oxidative stress and inflammatory responses (19, 20). Ethanol-induced gastric injury is an important experimental model that is widely used for preclinical evaluation of drugs with potential anti-gastric ulcer activity (21). In this study, we observed that a spaceflight mutant *L. reuteri* F-9-35 pretreatment markedly attenuated ethanol-induced gastric injury by inhibiting the major biomarkers of oxidative stress and inflammatory response, but not milk alone or the wild type. The results indicated that the gastroprotective activity of *L. reuteri* was strain-dependent. Our previous study suggested that probiotic characteristics of the mutant were superior to those of its wild type, including gastrointestinal resistance and adhesion (12). These characteristics may help the mutant colonize gastric mucosa after inoculation, and the colonized mutants act as a bacterial barrier to protect the mucosa from ethanol damage. Furthermore, the reduction of *Lactobacillus* in the gastric mucosa may be a contributing factor for gastric ulcer (22). Collectively, our results suggested that supplementation of *Lactobacillus* with high colonization ability in gastric mucosa is likely to have anti–gastric ulcer activity.

Mucus is an important defense barrier of the gastric mucosa against injury (23). Ethanol treatment causes mucus depletion of the gastric mucosal tissues (24), which was consistent with our results. In this study, pretreatment with *L. reuteri* F-9-35 can protect the stomach from ethanol damage by inhibiting the depletion of mucus in gastric mucosa, which may be a result of the increased activity of galactosyltransferase, a key enzyme in mucus synthesis (25). Moreover, bioactive NO in the gastric lumen is regarded as a key defense factor that protects the gastric mucosa from noxious agents (26). Decrease of gastric blood flow is one cause of gastric mucosa injury induced by ethanol (27). However, NO can inhibit platelet aggregation and thrombosis, enhance the blood flow of gastric mucosa and cell regeneration, and improve mucosal repair (28). A higher level of NO in the stomach from the F935 group was observed in our study, which indicated that *L. reuteri* F-9-35 pretreatment protects gastric mucosa from ethanol damage partly through improving gastric microcirculation.

Oxidative stress plays a significant role in the pathogenesis of gastric injury induced by ethanol (29). Ethanol administration causes the overproduction of reactive oxygen species (ROS) and acceleration of lipid peroxidation to aggravate gastric injury (30). Because ROS could convert unsaturated fatty acids to MDA through lipid peroxidation, MDA is usually used as a marker of lipid peroxidation (31). GSH is one of the intracellular antioxidants against ROS attacks. Once GSH is depleted, gastric tissue is more vulnerable to ethanol-induced oxidative injury (14). NO has also been proved to be an antioxidant that can accelerate gastric ulcer healing (32). In our study, *L. reuteri* F-9-35 inhibited MDA increase and improved the reduction of GSH and NO induced by ethanol. These results indicated *L. reuteri* F-9-35 inhibited ethanol-induced oxidative stress, which likely contributes to its gastroprotective action.

Previous studies reported that the higher expressions of some pro-inflammatory genes, such as TNF-α, IL-1β, and COX-2, were observed in gastric tissue after the intake of ethanol (33, 34), which was consistent with our results. TNF-α is a potent pro-inflammatory cytokine that activates neutrophil infiltration to cause the disturbance in gastric microcirculatory, thereby aggravating gastric injury (30). Increased IL-1β could recruit TNF-α and PG in the process of gastric injury induced by ethanol (35). The overexpression of IL-1β and TNF-α in gastric tissue could increase the crucial inflammatory factor COX-2, which converts PG to oxidation products involved in inflammatory response (36). Additionally, NF-κB is the classic pro-inflammatory transcription factor, which plays an important role in the inflammatory response of gastric tissue (34, 37). Activation of the NF-κB signaling pathway can induce a large number of inflammatory genes, including TNF-α, IL-1β, and COX-2 (38). In the present study, pretreatment with *L. reuteri* F-9-35 inhibited the increase of these inflammatory markers and decreased NF-κB activity. These findings indicated the anti-inflammatory action of *L. reuteri* F-9-35 against ethanol-induced gastric injury, which may be mediated by the inhibition of NF-κB activation.

In the present study the mechanism for how *L. reuteri* F-9-35 attenuated ethanol-induced gastric injury in rats was investigated by detecting some biomarkers of oxidative stress and inflammatory response. However, the pathophysiological mechanisms for ethanol-induced gastric ulcer are very complicated. The mechanism may involve gastric mucosal epithelial cell apoptosis, intercellular junction disorders, and alterations in epithelial transport, etc. (39, 40). These factors may be potential targets of *L. reuteri* F-9-35 against gastric ulcer, but further studies are needed. Additionally, it is still uncertain whether it is some metabolites of *L. reuteri* F-9-35 such as exopolysaccharides that contribute to the gastroprotective effect.

Several researches have reported that acute ethanol administration could damage the liver, resulting in dysfunction reflected by increased lipoperoxidation (MDA as a marker) and TG and decreased GSH in animals (41, 42). In this study, the levels of hepatic MDA, TG, and GSH in rats were also detected after consumption of ethanol. However, we observed that ethanol treatment did not cause changes in MDA level. Hashimoto and Recknagel (43) reported that ethanol did not increase the hepatic MDA level in rats at any time after binge time, which was consistent with our results. The sole determination of the MDA level is probably not suitable for evaluating hepatic lipid peroxidation in acute ethanol toxicity (44). Additionally, ethanol did not result in elevated TG and decreased GSH in this study, which may be a result of the short alcohol exposure time. Interestingly, pretreatment with *L. reuteri* F-9-35 enhanced the hepatic GSH level, which indicated the strain may have the potential to protect the liver from oxidative damage. Further study is needed to evaluate the hepatoprotective effect of *L. reuteri* F-9-35 in a model of ethanol-induced liver injury *in vivo*.

## Conclusion

In conclusion, a spaceflight mutant *L. reuteri* F-9-35 significantly relieved ethanol-induced gastric injury in rats compare to its wild type. This beneficial effect may be a result of a reduction in inflammatory response and oxidative stress by improving mucus secretion and the biosynthesis of GSH and NO, reducing the levels of MPO and MDA, downregulating the expressions of pro-inflammatory genes (TNF-α, IL-1β, and COX-2 mRNA) and inhibiting NF-κB activation in gastric tissue. Further efforts are needed to find variations of genome, proteome, and metabolome between *L. reuteri* F-9-35 and its wild type in order to understand the mechanisms for the different effects of the two strains on anti–gastric ulcer activity.
